# Intrathecal morphine versus epidural ropivacaine infusion for analgesia after Cesarean section: a retrospective study

**DOI:** 10.1186/s40981-015-0005-6

**Published:** 2015-08-27

**Authors:** Hiroko Suzuki, Yoshinori Kamiya, Takashi Fujiwara, Takayuki Yoshida, Misako Takamatsu, Kazunori Sato

**Affiliations:** 1 Division of Anesthesiology, Niigata University Graduate School of Medical and Dental Sciences, 1-757 Asahimachi-dori, Chuo Ward, Niigata, Niigata 951-8510 Japan; 2 Department of Anesthesia, Nagaoka Chuo General Hospital, 2041 Kawasaki-cho, Nagaoka, Niigata 940-8653 Japan; 3 Present address: Department of Anesthesiology, Uonuma Institute of Community Medicine, Niigata University Medical and Dental Hospital, 4132 Urasa, Minami-uonuma, Niigata 949-7302 Japan

**Keywords:** Cesarean delivery, Spinal anesthesia with intrathecal morphine, Opioid-free epidural analgesia, Postoperative pain, Adverse effects

## Abstract

**Background:**

Analgesia after Cesarean delivery (CD) requires early ambulation to prevent thromboembolic disease and to facilitate baby care. We retrospectively reviewed anesthesia charts and medical records of patients who underwent CD to compare the efficacy of spinal anesthesia supplemented with intrathecal morphine hydrochloride (ITM) and combined spinal–epidural anesthesia followed by opioid-free epidural analgesia (CSEA-EDA).

**Findings:**

All subjects underwent CD at Nagaoka Chuo General Hospital between February 2012 and January 2013. Patient characteristics, time to first analgesic rescue after CD, and analgesic use after CD were examined. Incidences of postural hypotension, lower extremity numbness/weakness, postoperative nausea/vomiting (PONV), and pruritus were also examined for 48 h after CD. Average time to first analgesic use after CD (ITM 25.13 ± 16.07 h, CSEA-EDA 22.42 ± 16.27 h, *p* = 0.521) and cumulative probability of rescue analgesic use (*p* = 0.139 by log-rank test) were comparable between groups. However, average analgesic use within 24 h was lower in the ITM group (0.75 ± 1.05 times) than in the CSEA-EDA group (1.52 ± 1.72 times, *p* = 0.0497). Numbness or motor weakness in lower extremities only occurred in the CSEA-EDA group, and pruritus only occurred in the ITM group.

**Conclusions:**

The results of this study suggest that ITM is better than CSEA-EDA for anesthesia following CD with regard to pain control. Also, ITM would be advantageous for early ambulation following CD because of lower incidence of numbness and motor weakness in lower extremities compared to CSEA-EDA.

## Findings

### Introduction

A Cesarean section is one of the most commonly performed surgical procedures. Combined spinal–epidural anesthesia (CSEA) is a favorable neuraxial anesthetic technique for Cesarean deliveries (CD) because the epidural catheter can also be utilized for postoperative patient-controlled epidural analgesia. However, postoperative epidural analgesia frequently causes lower extremity numbness and weakness, which can delay early ambulation [1, 2]. Opioid-supplemented epidural analgesia after CD can provide superior analgesia and has a low incidence of lower extremity numbness and weakness [3, 4]. However, opioid supplementation in continuous epidural analgesia can lead to postoperative nausea and vomiting (PONV), which hinders early ambulation and recovery. Moreover, in the perinatal period, women are more susceptible to thromboembolic disease, and perioperative anticoagulant therapy is recommended to prevent thromboembolic disease during pregnancy. Unfortunately, anticoagulants can make epidural catheter handling more difficult.

Morphine-supplemented spinal anesthesia [5], or single-shot intrathecal morphine (ITM), can rapidly induce anesthesia and provide long-term postoperative pain relief for elective and emergency CDs. Therefore, ITM may allow for early ambulation after CD, which reduces the risk thromboembolic disease and facilitates baby care. Unfortunately, intrathecal opioids are associated with PONV, pruritus, sedation, and respiratory depression [5, 6].

Numerous clinical studies have evaluated the efficacy of ITM and CSEA followed by epidural analgesia in patients undergoing CD. To the best of our knowledge, no study has directly compared anesthesia efficacy and adverse effect incidences between ITM and CSEA with opioid-free epidural analgesia (CSEA-EDA) within 48 h of CD. We retrospectively examined and compared the efficacy and safety of spinal anesthesia with ITM and CSEA-EDA after CD.

### Methods

#### Study subjects

This retrospective cohort study was performed at Nagaoka Chuo General Hospital. The study was reviewed and approved by the Nagaoka Chuo General Hospital institutional review board. Anesthesia charts and medical records of consecutive patients who underwent CD between February 2012 and January 2013 were reviewed. Patients that underwent spinal anesthesia without ITM or general anesthesia for CD were excluded from analyses.

#### Analgesic methods

The ITM group received 0.5 % hyperbaric bupivacaine (8.5–12 mg) supplemented with 100 μg of intrathecal preservative-free morphine hydrochloride. The CSEA-EDA group had an epidural catheter inserted at the T11/12, T12/L1, or L1/2 intervertebral space using standard procedures. After test dose administration (3 mL bolus of 1 % mepivacaine), 0.5 % hyperbaric bupivacaine (3.5–9.0 mg) was administered into the intrathecal space. Epidural 0.75 % ropivacaine (5–12 mL) was given as needed to obtain a block of adequate height. Immediately after surgery, 0.2 % ropivacaine was administered through the epidural catheter (4 mL/h) using a disposable infuser (Coopdeck Baloonjector 300 with PCA apparatus, Daiken Medical, Osaka, Japan). The CSEA-EDA group had anesthesia administered by one physician (KS), and the ITM group had anesthesia administered by two physicians (HS or TF).

All patients were mobilized 6–8 h after surgery. Based on pain level and patient requests, rescue analgesics were administered to manage postoperative breakthrough pain (numeric rating scale [NRS] ≥ 3–4). Patients were first administered suppository diclofenac sodium (50 mg) or oral loxoprofen sodium (60 mg). The CSEA-EDA group had the option of patient-controlled epidural anesthesia (PCEA; 0.2 % ropivacaine 3 mL, lockout interval of 60 min) for initial rescue analgesic administration. If pain persisted, intramuscular pentazocine (30 mg) was given. Acetaminophen (400 mg) was used as an alternative or supplementation to NSAID analgesia, if needed.

Data were collected within 48 h of CD and included patient characteristics, time to first rescue analgesic use, rescue analgesic use frequency, and adverse event (postural hypotension, lower extremity numbness and weakness, PONV, and pruritus) incidences. Primary outcomes included time to first rescue analgesic use and rescue analgesic use frequency. Secondary outcomes were the incidences of adverse effects within 48 h of CD.

#### Data analyses

Continuous variables are presented as mean ± standard deviation. Categorical variables, ordinal variables, and non-normally distributed data are presented as median (range). All statistical analyses were performed using Microsoft Excel 2011 for Macintosh (Microsoft, Redmond, WA, USA) with a statistical macro (XLSTAT 2014; Addinsoft, New York, NY, USA). An unpaired *t* test or Mann–Whitney *U*-test was used to determine statistical significance of differences in patient characteristics. A log-rank test was used to compare cumulative probabilities. A Kolmogorov–Smirnov test was used to compare time distribution of rescue analgesic use. Patients who did not require rescue analgesics were assigned a first use time of 48 h. The comparisons of frequency of rescue analgesic use within 48 h after CD and the average time to first rescue analgesic use between groups in which the epidural puncture was performed were made using Kruskal–Wallis test and one-way analysis of variance (ANOVA), respectively. The comparisons between groups in which epidural analgesia was continued or discontinued within 48 h after CD were performed using Mann–Whitney *U*-test and unpaired *t* test, respectively. Incidences of anesthesia-related adverse effects were compared using Fisher’s exact tests. Statistical significance was defined as *p* < 0.05.

### Results

**Fig. 1:**
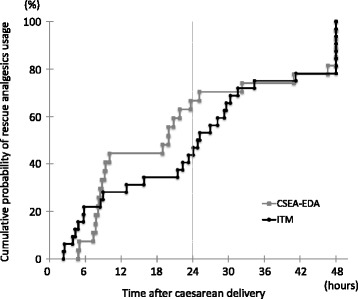
Cumulative probability of rescue analgesic use after Cesarean delivery. There was no significant difference in the cumulative probability of rescue analgesic use within 48 h of Cesarean delivery in patients receiving single-shot spinal analgesia (ITM group) and combined spinal–epidural anesthesia followed by opioid-free epidural analgesia (CSEA-EDA group, *p* = 0.138 by log-rank test)

**Fig. 2:**
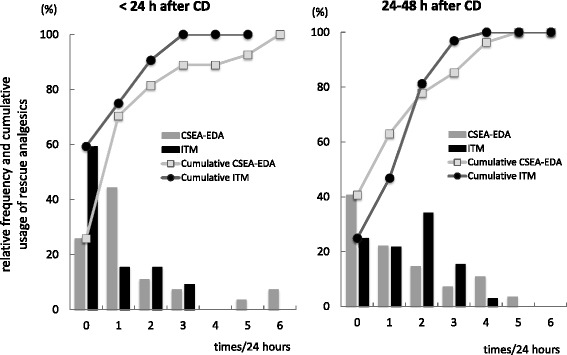
Relative frequency of rescue analgesic usage within 24 h of Cesarean delivery (CD) and between 24 and 48 h after CD in patients receiving single-shot spinal analgesia (ITM group) and combined spinal–epidural anesthesia followed by opioid-free epidural analgesia (CSEA-EDA group). Within 24 h of CD, the relative frequency and distribution of rescue analgesic use in the ITM group (ITM 0 times [range 0–3]) was lower than that in the CSEA-EDA group (1 time [0–6], *p* = 0.0497 by Mann–Whitney *U*-test, *p* = 0.019 by Kolmogorov–Smirnov test). Rescue analgesic use between 24 and 48 h after CD was not significantly different between groups (ITM 2 times [0–4], CSEA-EDA 1 time [0–5], *p* = 0.416 by Mann–Whitney *U*-test, *p* = 0.465 by Kolmogorov–Smirnov test). *Vertical bar charts* represent the relative frequency of rescue analgesic use in each group. *Line plots* represent the cumulative frequency of rescue analgesic use in each group. Data are presented as median [range]

Eighty-five patients underwent CDs which occurred during the study period, of which 32 and 27 patients were included in the ITM and CSEA-EDA groups, respectively. Patient demographic characteristics are summarized in Table 1. No significant differences between groups were observed in age, height, body weight, gestational week, and emergency CD rate. The number of cases per site of epidural puncture in CSEA procedure was as follows: T11/12, *n* = 8; T12/L1, *n* = 12; and L1/2, *n* = 7. Average time to first rescue analgesic use (including patient-controlled local anesthetic bolus infusion in the CSEA-EDA group) was not statistically different between groups (ITM 25.13 ± 16.07 h, CSEA-EDA 22.42 ± 16.27 h, *p* = 0.521, unpaired *t* test). Moreover, the cumulative probability of rescue analgesic and/or PCEA use was not significantly different between groups (Fig. 1). The percentage of patients using rescue analgesics within 48 h of CD was similar between groups (ITM 25 of 32 patients [78.1 %], CSEA-EDA 22 of 27 patients [81.5 %]). The frequency of rescue analgesic use was significantly lower in the ITM group (0 time [range 0–3]) than in the CSEA-EDA group (1 time [0–6], *p* = 0.0497) within 24 h of CD, but not in the 24–48 h after CD (ITM 2 times [0–4], CSEA-EDA 1 time [0–5], *p* = 0.465; Fig. 2). The frequency of rescue analgesic use within 48 h of CD and the average time to first rescue analgesic use were not significantly different between the groups in which the epidural puncture was performed (Table 2).

**Table 1:** Demographic data of the patients included in this study

	ITM group (*n* = 32)	CSEA-EDA group (*n* = 27)	*p*-value
Age (years)	33.6 ± 5.2	31.4 ± 4.1	0.24
Height (cm)	158 ± 5.3	157 ± 4.1	0.30
Weight (kg)	64.3 ± 9.3	63.4 ± 8.9	0.62
Gestational week	38.0 ± 1.7	38.5 ± 2.1	0.55
Emergency CD incidence	11 (34 %)	12 (44 %)	0.30

Data are shown as mean ± SD or as actual incidence number (percentage of total patients). An unpaired *t* test was used to analyze the age, height, weight, and gestational week; Fisher’s exact test was used to analyze the incidences of emergency CD

*ITM* intrathecal morphine-supplemented spinal anesthesia, *CSEA-EDA* combined spinal–epidural anesthesia followed by opioid-free epidural analgesia, *CD* Cesarean delivery

**Table 2:** Frequency of rescue analgesic use and average time to first rescue analgesic use within 48 h after CD between the groups, in which the epidural puncture was performed

		T11/12	T12/L1	L1/2	*p*-value
		(8/27; 29.6 %)	(12/27; 44.4 %)	(7/27; 25.9 %)	
Frequency of rescue analgesic use after CD (times)	<24 h	1 [0–6]	0 [0–3]	1 [0–5]	0.59
	24–48 h	0 [0–3]	1 [0–4]	2 [0–5]	0.09
	<48 h	1 [0–9]	2 [0–6]	4 [1–11]	0.1
Average time to first rescue analgesic use (hh:mm)		28:47 ± 17:51	19:12 ± 16:42	20:38 ± 13:37	0.43

Data are shown as median [range] or mean+/-SD. Statistical analyses were performed by Kruskal–Wallis test for frequency of rescue analgesic use and by one-way ANOVA for average time to first rescue analgesic use within 48 h after CD

*CD* Cesarean delivery

Analgesia-related adverse effects occurred within 48 h in both groups. Lower extremity numbness and weakness only occurred in the CSEA-EDA group, and pruritus only occurred in the ITM group (Table 3). The incidence of numbness or motor weakness in CSEA-EDA group was as follows: T11/12, *n* = 2; T12/L1, *n* = 4; and L1/2, *n* = 3. The odds ratio of incidence of numbness or motor weakness was not significantly different between the groups in which the epidural puncture was performed. Postural hypotension occurred more often in the CSEA-EDA group, but the difference was not statistically significant (*p* = 0.08). Five of 27 patients (18.5 %) in the CSEA-EDA group had epidural catheter issues (i.e., connector trouble or local anesthetic leakage from catheter insertion site). Catheter issues led to obstetrician removal of the catheter within 48 h of CD in 12 of 27 patients (44.4 %) in the CSEA-EDA group. The frequency of rescue analgesic use within 24 h of CD and the average time to first rescue analgesic use were not significantly different between groups that discontinued epidural analgesia within 48 h of CD and those that continued analgesia (Table 4). Respiratory depression was not observed in any patient in either group.

**Table 3:** Incidence of adverse effects associated with each anesthetic technique

	ITM group (*n* = 32)	CSEA-EDA group (*n* = 27)	*p*-value
Postural hypotension	2 (6.3 %)	6 (22.2 %)	0.08
PDPH	–	2 (7.4 %)	0.21
Numbness in the lower extremities 24 h after CD	–	9 (33.3 %)	<0.001
PONV	2 (6.3 %)	2 (7.4 %)	0.63
Pruritus	12 (37.5 %)	–	<0.001
Catheter trouble	–	5 (18.5 %)	–

Data are shown as actual incidence number (percentage of total patients). Statistical analyses were performed by Fisher’s exact test

*CD* Cesarean delivery, *ITM* intrathecal morphine-supplemented spinal anesthesia, *CSEA-EDA* combined spinal–epidural anesthesia followed by opioid-free epidural analgesia, *PDPH* postdural puncture headache, *PONV* postoperative nausea and vomiting

**Table 4:** Frequency of rescue analgesic use and average time to first rescue analgesic use within 48 h after CD between the groups, in which the postoperative epidural analgesia was continued for 48 h or was discontinued

		Continued epidural analgesia	Discontinued epidural analgesia	*p*-value
		(15/27; 55.6 %)	(12/27; 44.4 %)	
Frequency of rescue analgesic use after CD (times)	<24 h	1 [0–6]	1 [0–6]	0.5
	24–48 h	0.5 [0–4]	2 [0–5]	0.2
	<48 h	1.5 [0–9]	2 [0–11]	0.61
Average time to first rescue analgesic use (hh:mm)		21:16 ± 14:33	23:28 ± 18:12	0.73

Data are shown as median [range] or mean+/-SD. Statistical analyses were performed by Mann–Whitney *U*-test for frequency of rescue analgesic use and by unpaired *t* test in average time to first rescue analgesic use within 48 h after CD

*CD* Cesarean delivery

### Discussion

This small retrospective study revealed that the ITM group required fewer analgesic interventions than the CSEA-EDA group within 24 h of CD. Additionally, fewer adverse effects that impeded early ambulation (lower extremity numbness and weakness, orthostatic hypotension) occurred in the ITM group than in the CSEA-EDA group.

Intrathecal opioids are effective for managing postoperative pain after CD but are associated with pruritus, PONV, and respiratory depression [5–7]. Lower extremity numbness more than 24 h after CD and respiratory depression were not observed in the ITM group. Additionally, PONV incidence in the ITM group was not statistically different than in the CSEA-EDA group. Previous studies have shown that respiratory depression is very rare in patients undergoing CD with morphine-supplemented spinal anesthesia [8]. Moreover, 0.1 mg of morphine sufficiently reduces postoperative pain with little respiratory depression [5, 9].

Previous studies demonstrated that CSEA-EDA adequately manages postoperative pain after CD and has a low incidence of adverse effects (PONV and hypotension) [10, 11]. However, early ambulation after CD is important to prevent thromboembolic diseases and to facilitate infant breastfeeding and care. Therefore, postoperative epidural analgesia may be disadvantageous because epidural analgesia with local anesthetics causes lower extremity numbness and weakness. Previous reports found lower extremity weakness incidences of 26 % [3] and 11–41 % [11] after CD and 11.5 % after gynecological and urological surgery [12] when postoperative epidural analgesia was used. Furthermore, lower extremity numbness incidences were 42 % after gynecological surgery [13], 37.5–67.5 % after CD [4], and 35.5 % after gynecological and urological surgeries [12] when postoperative epidural analgesia was used. In our study, numbness or motor weakness of lower extremities occurred in 33.3 % of patients in the CSEA-EDA group, and the site of epidural catheter insertion was not correlated with this adverse effect. This result is comparable to that of the previous studies listed above. In most cases, CSEA was performed with CSEA needle and puncture site was L2/3–L4/5. In such cases, incidence of numbness or motor weakness of lower extremities would be expected to be much higher than that observed in our study.

In this study, none of the patients in the CSEA-EDA group were converted to general anesthesia or to intraoperative supplemental administration of local anesthetics. Therefore, we concluded that the patients in both groups received adequate anesthesia for CD. In addition, although the average dose of spinal bupivacaine in the CSEA-EDA group was significantly lower than that in the ITM group, as shown in *Anesthetic Methods* (ITM group 9.75 ± 0.87 mg, CSEA-EDA 5.42 ± 0.97 mg, *p* < 0.001, Mann–Whitney *U*-test), the average time to first rescue analgesic use in the CSEA-EDA group was comparable to that in the ITM group. These results suggested that epidural anesthesia was effective for managing postoperative pain after CD. There was no statistical significance in analgesic efficacies between different intervertebral spaces of epidural puncture site.

Moreover, we also found that 44 % of CSEA-EDA patients had discontinued epidural analgesia within 48 h of CD because of lower extremity numbness and weakness (33.3 %) and/or catheter trouble (18.5 %). When epidural analgesia is discontinued, another rescue analgesic is needed to manage postoperative pain. However, analgesic efficacies within 24 h of CD were not significantly different between the groups that discontinued epidural analgesia within 48 h of CD and those that continued analgesia. Postoperative analgesia in the early period after CD might be crucial for postoperative pain control.

Though pruritus is not life-threatening, it can severely impact recovery quality in CD patients administered with opioids. Pruritus only occurred in the ITM group with a similar incidence as previously reported [14–16]. Unfortunately, our retrospective data do not allow us to comment on how pruritus affected postpartum recovery.

### Conclusions

This retrospective cohort study had several limitations. First, patient pain was not evaluated using a subjective scale because this information was not in medical records. Therefore, postoperative pain was evaluated with supplemental analgesic use. Second, retrospective studies carry inherent biases because they are not randomized or blinded. Lastly, our study included a relatively small number of subjects.

In conclusion, our study suggests that ITM is as effective as CSEA-EDA in managing postoperative pain after CD, but ITM does not inhibit early ambulation. Unfortunately, pruritus can occur with ITM, and countermeasures to prevent this should be considered.

## Abbreviations

CD: Cesarean delivery
CSEA: combined spinal–epidural anesthesia
EDA: epidural analgesia
ITM: intrathecal morphine
PONV: postoperative nausea and vomiting
